# Compliance with the 24-h Movement Guidelines for Portuguese children: differences between boys and girls

**DOI:** 10.1038/s41598-023-49227-6

**Published:** 2024-05-24

**Authors:** João Martins, Miguel Ángel Tapia-Serrano, Telma Nogueira, Pedro Antonio Sanchéz-Miguel

**Affiliations:** 1 Centro de Estudos em Educação, Faculdade de Motricidade Humana e UIDEF, Instituto de Educação, Universidade de Lisboa, 1649-004 Lisbon, Portugal; 2 Departamento de Didáctica de la Expresión Musical, Plástica y Corporal, Grupo Análisis Comportamental de la Actividad Física y el Deporte (ACAFYDE), Facultad de Formación del Profesorado, Universidad de Extremadura, Av. de la Universidad, S/N, 10004 Cáceres, Spain; 3 Instituto de Saúde Ambiental, Faculdade de Medicina, Universidade de Lisboa, 1649-028 Lisbon, Portugal; 4 Laboratório de Nutrição, Faculdade de Medicina, Universidade de Lisboa, 1649-028 Lisbon, Portugal; 5 Departamento de Didáctica de la Expresión Musical, Plástica y Corporal, Grupo de Investigación Análisis Didáctico y Comportamental del Deporte (ADICODE), Facultad de Formación del Profesorado, Universidad de Extremadura, Av. de la Universidad, S/N, 10004 Cáceres, Spain

**Keywords:** Risk factors, Psychology, Human behaviour, Epidemiology

## Abstract

The 24-h Movement Guidelines for children recommend at least 60 min per day of moderate-to-vigorous intensity physical activity (PA), ≤ 2 h/day of screen time, and 9–11 h/day of sleep for children. Since little information is available on the 24-h Movement Guidelines in Portuguese children, this research attempts to know the proportion of Portuguese children meeting individual and combinations of these 24-h Movement Guidelines. Another aim of this study is to test sex differences in compliance with 24-h Movement Guidelines. A final sample of 1351 Portuguese children (51.4% girls, aged 7.65 ± 1.21) participated in this cross-sectional study. PA, screen time and sleep duration were parent-reported through a questionnaire. Only 3.7% of children met all three 24-h Movement Guidelines, whereas 11.9% met none. Although boys showed greater compliance with PA recommendations and girls with screen time recommendations, no significant sex differences were found in the compliance of all three 24-h Movement Guidelines. Given that 96.3% of Portuguese children did not meet 24-h Movement Guidelines, promoting these three movement behaviours in both boys and girls is crucial to encouraging positive consequences early.

## Introduction

It is well documented that meeting recommendations for physical activity (PA)^1^, recreational screen time^2^ and sleep duration are independently associated with positive health benefits in young people. Although these behaviours have generally been studied in isolation, there is compelling evidence that they are co-dependent because they are distributed throughout the day (24-h period). In 2016, Canadian experts developed the 24-h Movement Guidelines for children and adolescents (aged 5–17)^3^. These guidelines recognise that the whole day is essential and that individual behaviours such as PA, screen time and sleep duration should be considered simultaneously^4^.

According to the 24-h Movement Guidelines, in 24 h, children aged 5 to 13 years should accumulate at least 60 min per day of moderate-to-vigorous PA, ≤ 2 h/day of recreational screen time and 9–11 h of sleep per day (for children aged 5 to 13 years old)^4^. Adhering to all three 24-h Movement Guidelines has been related to more physical, social and cognitive health benefits than adhering to only one or none of these recommendations^5^. Furthermore, meeting the 24-h Movement Guidelines in middle adolescence has also been associated with a lower potential risk for type 2 diabetes mellitus in adulthood^6^.

Despite the benefits, a systematic review and meta-analysis involving 387,437 young people worldwide from 23 different countries revealed that only 7.12% of individuals adhered to the three 24-h Movement Guidelines. Specifically, only 10.3% of children met all the 24-h Movement Guidelines, with adherence significantly lower in girls (3.8%) than boys (6.9%). Conversely, adherence to none of the three 24-h Movement Guidelines in children was 15.6%, with higher values in girls compared to boys^7^. This is a concerning result since non-compliance with the three 24-h Movement Guidelines has been associated with worse consequences on physical, social, and cognitive healt^5,6^.

In Europe, only 9.6% of young people met all three 24-h Movement Guidelines, while 13.5% did not meet any recommendations^7^. To our knowledge, only two studies have analysed the proportion of Portuguese preschool children and children meeting the 24-h Movement Guidelines^8,9^. In the study involving preschool children up to 5 years old, where PA was accelerometry-derived, and screen time and sleep duration were parent-reported, it was found that only 4.5% of participants complied with the 24-h Movement Guidelines. In contrast, 11.1% did not meet any guidelines^9^. In another study involving 639 Portuguese children aged 9–12 years^8^, PA and sleep duration were assessed using accelerometry, while screen time was self-reported. The study showed that only 2.0% of the participants met the 24-h Movement Guidelines, while 28.5% did not meet any of these recommendations^8^. These findings cannot be generalised to the Portuguese children, as they only included small and non-representative samples of preschool children and children from the Porto region^8,9^. Due to these limitations, more studies examining the prevalence of the 24-h Movement Guidelines among Portuguese children are needed, mainly in the ages of 6 to 9 years, where no data is available. Another limitation of the study conducted by Román-Viñas et al.^8^ was that they did not analyse adherence to the three 24-h Movement Guidelines in boys and girls separately. Given that adherence to these recommendations may differ between sexes^5^, it is suggested that the prevalence of the 24-h Movement Guidelines should be tested independently for boys and girls.

Therefore, considering the above limitations, this study aims to: (1) identify the proportion of Portuguese children who individually and in combination comply with the three 24-h Movement Guidelines; and (2) examine the differences between boys and girls in compliance with the 24-h Movement Guidelines.

## Methods

### Design and participants

This cross-sectional study was conducted in Sintra Municipality, Portugal, within the scope of the *Sintra Grows Healthy* (SGH) project^10^. The SGH is a school-based intervention to promote healthy lifestyles related to nutrition and PA among primary schoolchildren. The SGH has been developed by the Sintra Municipality in partnership with health, education and academic entities as stakeholder^10^. SGH has the institutional support of organisations such as the Ministry of Health, the Portuguese National Programme for the Promotion of Healthy Eating, the Portuguese National Programme for the Promotion of Physical Activity, and the Ministry of Education^10^. The Faculty of Human Kinetics is the academic partner leading the PA promotion within the SGH.

Sintra is the second Portuguese municipality with the most inhabitants under 15—67,222 in 2021^11^. Sintra municipality has 20 public school clusters, including 83 primary schools and 13,000 students. Initially, only a few schools were selected at their convenience to participate in the SGH. Every year, new schools are involved in the project, with the SGH consortium aiming to reach all the schools in Sintra during the next years^10^.

Considering the aims of the present study, the sample includes data from all children who participated in SGH in the 2020/2021 school year, regardless of their status in the SGH (intervention group or control group). In the 2020/2021 school year, 17 schools participated in the SGH study. A total of 2060 children out of 3907 had the legal written guardian’s consent and participated in the study. No child refused to participate during the study. Data collection took place between October and November 2020.

Any children who met the inclusion criteria and did not meet the exclusion criteria were considered to participate in the study. The inclusion criteria were as follows: (1) attend the primary schools belonging to the project; (2) the children’s legal guardian provide their written freely, enlightened, informed consent for participation. The exclusion criteria were as follows: (1) having a motor/intellectual disability; and/or (2) not having a valid consent for participation. The present study has been conducted following the Helsinki ethical guidelines for conducting research with humans and adding the approval from the Lisbon Academic Medical Centre ethics committee (401/17), the National Data Protection Commission (11468/2017) and the Ethics Boards of each participating school. The SGH study did not represent any risk, cost, or harm to the participants. Children’s participation was voluntary, and any child could refuse to participate at any moment in the study.

### Measures

#### Physical activity

The child’s legal guardian reported total PA by asking: “*In the last 7 days, how many days did your child practice physical activity for at least 60 min*?”. The answer options ranged from 0 to 7 days. This single-item valid and reliable question has been used in several epidemiological surveys in diverse cultures with children and adolescents^12,13^. Parent-report measures are accurate and reliable in assessing children’s PA^14,15^. In the present study, we used this single-item question for the parent to report their child’s overall PA. The child was considered to meet the PA guidelines^16^ if the answer was equal to 7 days.

#### Screen time

Based on Marshall et al.’s^17^ measures, the parents reported the time their child spent watching television, playing video games, using the smartphone, and using the computer during their leisure time on a regular weekday. The answer options for each behavior were: none, 15 min, 30 min, 1 h, 2 h, 3 h, 4 h, 5 h, and 6 h or more. These indicators have been used in a national representative survey^18–20^) in which parents reported their child’s screen time-related behaviours and in other surveys and studies^18,21^). Screen time-related behaviours were also parent-reported through questionnaires in one study focused on adherence to 24-h Movement Guidelines among Portuguese preschool children^9^. A new variable was created to analyse if children spent less than 2 h during leisure time and met the screen time guidelines by summing up the total time spent on these four screen-related behaviours^4^.

#### Sleep duration

Parents reported their child's usual sleeping hours per night on weekdays and weekends. These questions have been used in the Inquérito Alimentar Nacional e de Atividade Física (IAN-AF) survey for the parents of children aged 3–9 years old^19,20^. Daily sleep time was calculated by weighting weekday and weekend days using a ratio of 5:2 (e.g. [Daily sleep time on weekdays × 5] + [Daily sleep time on weekend days × 2]/7). Sleep duration guidelines were also calculated using the recommendations for children (9–11 h/day)^4^.

### Statistical analysis

Descriptive statistics were used to examine the average number of days spent in PA, screen time and sleep duration. Descriptive statistics were also used for identifying the proportion of participants meeting: (a) none of the 24-h Movement Guidelines; (b) PA only; (c) screen time only; (d) sleep duration only; (e) PA and screen time; (g) PA and sleep duration; (g) meeting screen time and sleep duration; and (i) all three recommendations. Differences between boys and girls for the 24-h Movement Guidelines were tested using the Student's t-test for continuous variables and the chi-square test for categorical variables. All analyses were performed with SPSS version 23.0 (IBM, Armonk, NY, USA), and the significance level was set at *p* < 0.05.

## Results

The descriptive characteristics of the participants and the prevalence of the three 24-h Movement Guidelines are shown in Table 1. 1351 Portuguese children (51.4% girls; 7.65 ± 1.21 years) participated in this cross-sectional study since they had valid data for all variables used. The average number of days per week usually engaged in 60 min of PA was 3.69. Average screen time and sleep duration were 2.10 h/day and 9.61 h/day, respectively. Although boys were more active than girls (*p* = 0.010), no significant sex differences were found for screen time (*p* > 0.05) and sleep duration (*p* > 0.05).

**Table 1:** Descriptive characteristics of the sample participants and prevalence of the three 24-h Movement Guidelines among boys and girls.

Study variables	Total	Boys	Girls	*p*
	M ± SD	M ± SD	M ± SD	
Sample *n* (%)	1351 (100.0)	656 (48.6)	695 (51.4)	
Age subgroups				**0.021**
5 years *n* (%)	15 (1.1)	4 (0.6)	11 (1.6)	
6 years *n* (%)	289 (21.4)	142 (21.6)	147 (21.2)	
7 years *n* (%)	303 (22.4)	126 (19.2)	177 (25.5)	
8 years *n* (%)	350 (25.9)	172 (26.2)	178 (25.6)	
9 years *n* (%)	346 (25.6)	185 (28.2)	161 (23.2)	
10 years *n* (%)	43 (3.2)	25 (3.8)	18 (2.6)	
11 years *n* (%)	5 (0.4)	2 (0.3)	3 (0.4)	
Age (years)^a^	7.65 ± 1.21	7.72 ± 1.23	7.57 ± 1.20	**0.026**
Physical activity (days/week)^a^	3.69 ± 1.66	3.81 ± 1.70	3.58 ± 1.61	**0.010**
0 days *n* (%)	105 (7.8)	43 (6.6)	62 (8.9)	
1 day *n* (%)	166 (12.3)	83 (12.7)	83 (11.9)	
2 days *n* (%)	452 (33.5)	201 (30.6)	251 (36.2)	
3 days *n* (%)	283 (20.9)	147 (22.4)	136 (19.6)	
4 days *n* (%)	155 (11.5)	80 (12.2)	75 (10.8)	
5 days *n* (%)	103 (7.6)	52 (7.9)	51 (7.3)	
6 days *n* (%)	24 (1.8)	12 (1.8)	12 (1.7)	
7 days *n* (%)	63 (4.7)	38 (5.8)	25 (3.6)	
Screen time (h/day)^b^	2.10 ± 1.68	2.15 ± 1.68	2.06 ± 1.67	0.305
Television (min/day)^b^	73.09 ± 57.87	71.55 ± 56.81	74.55 ± 58.86	0.402
Video games (min/day)^b^	29.44 ± 47.81	37.16 ± 53.45	22.17 ± 40.51	** < 0.001**
Smartphone (min/day)^b^	6.52 ± 26.13	4.05 ± 17.69	8.85 ± 31.96	** < 0.001**
Computer (min/day)^b^	17.04 ± 42.74	16.19 ± 42.73	17.85 ± 41.77	0.404
Sleep duration (h/day)^a,c^	9.61 ± 3.95	9.72 ± 4.57	9.50 ± 3.26	0.333
Sleep duration week day^b^	9.43 ± 4.76	9.73 ± 5.97	9.38 ± 4.05	0.230
Sleep duration weekend day^b^	9.67 ± 4.00	9.71 ± 4.26	9.74 ± 3.805	0.653

n, number of subjects; M, mean; SD, standard deviation.

Significant values are in bold.

^a^Tested with the Student’s T-test for independent samples.

^b^Tested with the Chi-square test.

^c^Sleep duration calculated by weighting weekday and weekend day using a ratio of 5:2.

**Figure 1:**
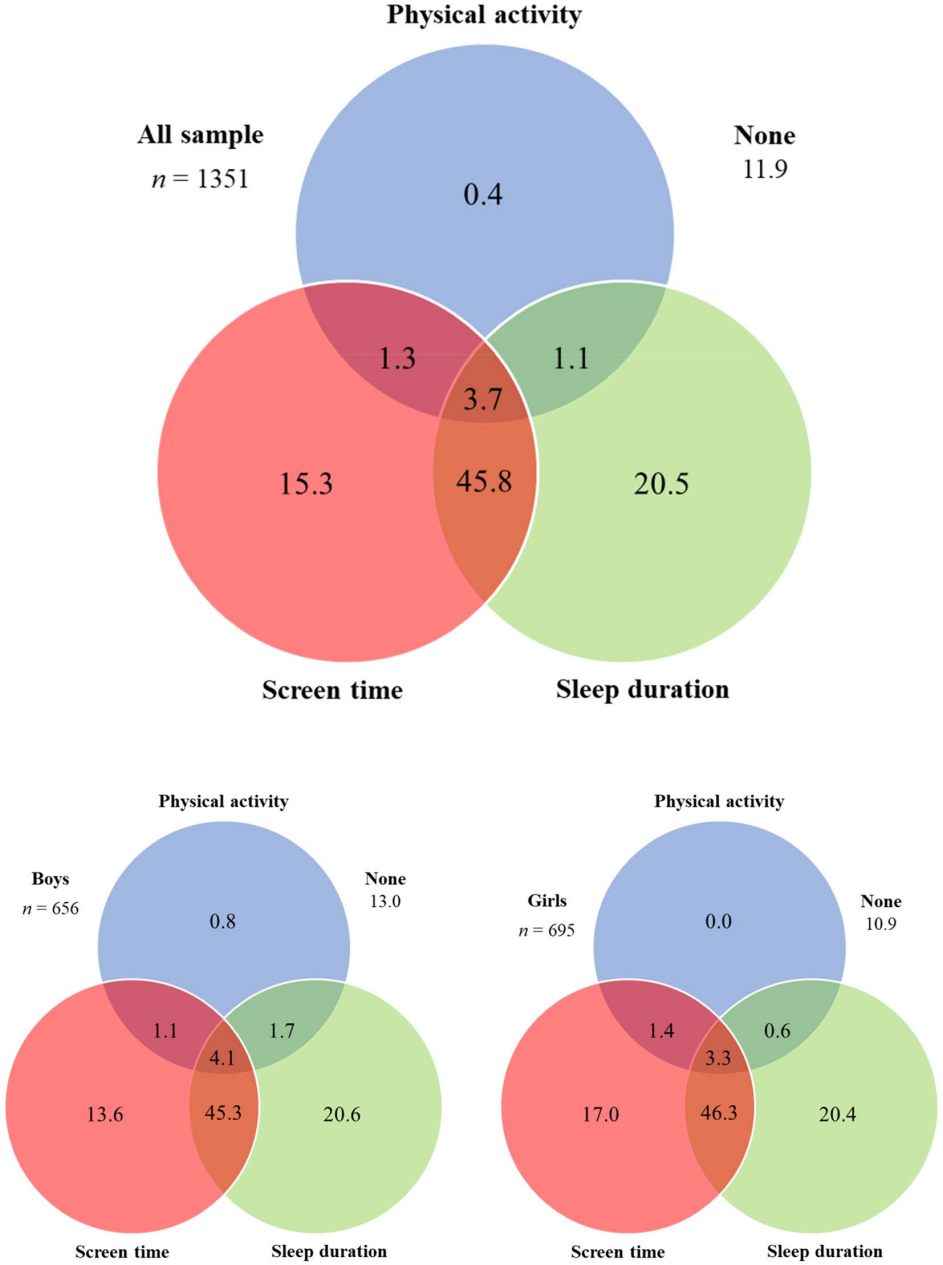
Venn diagram showing the proportion (%) of participants meeting 24-h Movement Guidelines separately and in all possible combinations*.*

Figure 1 shows the proportion of children meeting the 24-h Movement Guidelines separately and in all possible combinations. Only 3.7% of participants met all three 24-h Movement Guidelines, while 11.9% did not meet recommendations. Regarding independent compliance, 20.5% of children complied with the sleep duration guidelines, but only 15.3% and 0.4% met the screen time and PA recommendations, respectively. Concerning combined compliance, 45.8% of children met the screen time and sleep recommendations, only 1.3% met the PA and screen time recommendations, and 1.1% met the PA and sleep duration recommendations. In terms of sex, 4.1% of boys and 3.3% of girls met all three 24-h Movement Guidelines, while the prevalence of boys and girls who did not meet any of the recommendations was 13.0% and 10.9%, respectively.

Table 2 shows compliance with the 24-h Movement Guidelines between boys and girls. Overall, compliance (2.7% vs. 2.3) and non-compliance (13.0% vs. 10.9%) with the three 24-h Movement Guidelines was higher in boys than in girls. Nevertheless, the results revealed no significant differences between boys and girls in any comparisons (all, *p* > 0.05).

**Table 2:** Meeting of 24-h Movement Guidelines among boys and girls.

Meeting 24-h movement guidelines	Total	Boys	Girls	*p*
	*n* (%)	*n* (%)	*n* (%)	
Not meeting any guidelines	162 (11.9)	85 (12.9)	77 (11.0)	0.567
Meeting exclusively 1 guideline	491 (36.2)	230 (35.0)	261 (37.4)	0.339
Physical activity	87 (6.4)	50 (7.6)	37 (5.3)	0.085
Screen time	893 (66.1)	420 (64.0)	473 (68.1)	0.118
Sleep duration	961 (71.1)	470 (71.1)	491 (70.6)	0.686
Meeting exclusively 2 guidelines	653 (48.2)	316 (48.0)	337 (48.3)	0.904
Physical activity + screen time	67 (5.0)	34 (5.0)	33 (4.7)	0.433
Physical activity + sleep duration	65 (4.8)	38 (4.8)	27 (3.9)	0.935
Screen time + sleep duration	669 (49.5)	324 (49.4)	345 (49.6)	0.962
Meeting 3 guidelines				
Physical activity + screen time + sleep duration	50 (3.7)	27 (4.1)	23 (3.3)	0.604

n, number of subjects.

## Discussion

To the best of our knowledge, this is the first study that analysed the overall adherence to the 24-h Movement Guidelines among a sample of Portuguese children aged between 6 and 9 years and the differences between boys and girls. The main findings of the present study are as follows: (i) a total of 3.7% of children met all three 24-h Movement Guidelines; (ii) 11.9% of children met none of the three guidelines; (iii) no differences were found among boys and girls concerning the meeting of 24-h Movement Guidelines.

Considering that meeting the three guidelines has been associated with greater health-related, cognitive, and psychosocial benefits^5,6^, only 3.7% of children met the 24-h Movement Guidelines is of concern. These results are slightly lower than those obtained in previous studies^5,7,8^. For example, in an international context, a systematic review and meta-analysis included 63 studies from 23 countries, 10.3% of children met the 24-hs Movement Guidelines^7^, but a high variability of self-reported and device-based measures was shown. In the Portuguese context, in a study where the PA was accelerometry-derived and the screen time and sleep duration were reported by parents via questionnaire, only 4.5% of preschool children^9^ met all the 24-h Movement Guidelines. In another Portuguese study, where two of those behaviours were assessed using accelerometers (PA and sleep duration) and self-reported through a questionnaire (screen time), the prevalence of children (aged 10.5 years) adhering to the 24-h Movement Guidelines was even lower (2%)^8^.

The prevalence of children meeting the PA guidelines in our study was even lower than the frequent already lower rates found worldwide^22–24^. In Portugal, studies that are representative and have used parent-reported measures (such as diaries and/or questionnaires) also evidenced relatively higher PA levels among Portuguese children compared to those found in the present study^21,25^. These differences might be explained by how PA was measured (accelerometers, self-reported, parent-reported), the sample characteristics (representative vs convenience), and the time when the study occurred. Notably, the current study has baseline data collected between October and November of 2020, during the Covid-19 pandemic (outside of lockdown periods). The preliminary evidence during the covid19 period suggests a decrease in PA and increased sedentary behaviors among children^26^. Therefore, caution is recommended when interpreting these results.

In addition, to increase the children’s PA levels, it is crucial to consider the other two health-related behaviours, screen time and sleep. Previous studies, where screen-based sedentary behaviours were parent-reported, show evidence that these behaviors are concerningly high among children^21,25^. In a scoping review of 130 surveillance studies since 2000, Thomas et al.^27^ identified that about half of children and adolescents exceeded 2 h/day of total screen time. Indeed, total screen time was 3.6 h/day. In Portugal, data reported by parents through a questionnaire suggests that 36.5% of children aged 6–9 years spent more than 2 h/day watching TV^25^. This value increased to 71.3% of participants during the weekend day. In the study of Rito et al.^21^, parents reported that about 60% of children spent between 1 and 2 h playing video games daily. Reducing and limiting total screen time daily is critical to benefit children’s health^28,29^.

In the present study, most children met the sleep duration guideline of 9–11 h daily. This finding relates to a previous study involving children aged 6–8 years, where it was found that 96% slept 9 h or more^21^. These results are better than those presented by Roman-Viñas et al.^8^, who assessed the sleep duration using an accelerometer, where only 18.1% of children aged 9–12 years met the guidelines. This can be due, for example, to the different methods used to assess the sleep duration, the cutoff values, and the age in both samples. Despite the positive results, there is room for improvement. For example, quality sleep is of higher importance to prevent detrimental health outcome^30,31^ and future studies should consider this type of measure, in addition to sleep duration.

In the current study, 11.9% of children did not meet any of the three guidelines. In the systematic review conducted by Tapia Serrano et al.^7^ found about 15.5% of children in this condition. Since these children are not meeting any of the guidelines, they are at a higher health risk and, therefore, need to be considered as a priority group for interventions to increase adherence to 24-h Movement Guidelines.

In the present study, more boys (2.7%) met the 24-h Movement Guidelines than girls (2.4%), but the differences were not statistically significant. PA tends to be consistently lower in girls than in boys^24,25^, regardless of how this variable is assessed (e.g. self-reported or objectively measured). Still, this trend tends to be more evident during adolescence^13^. In another study, boys were more active and less sedentary than girls^22^. It can be hypothesised that these differences can be related to measurement issues, the sample characteristics, and the moment when the study occurred (i.e., amid the covid19 pandemic).

Other motives that explain the PA differences among boys and girls can be related to individual (e.g., age, self-efficacy), social (e.g., family support), and environmental factors^32^. Some studies also explored the correlates of meeting the 24-hs Movement Guidelines and found that, for example, being younger, being from a family with higher educational and socioeconomic levels, and not having a TV in the bedroom were positively correlated factors^33–36^. It has also been shown that the country's development index is strongly related to compliance with the 24-h Movement Guidelines^7^. Therefore, it could be expected that the differences between the results of the present study and previous research are the result or related to the country’s development index and socioeconomic and cultural characteristics. Further studies are needed to explore the correlates and determinants of children meeting the 24-h Movement Guidelines, worldwide and in Portugal.

This study has limitations and strengths. The cross-sectional design of the study precludes any causal inferences. The fact that the sample was obtained by convenience precludes the generalisation of the findings to the child population in Portugal. Adapted parent-reported questions were used in this study and may be susceptible to social desirability bias. Still, parent-reported measures in the 24-h Movement Guidelines study for children are common in the literature^7^. Further instruments are needed to enhance the knowledge of the 24-h Movement in the early ages^37^. We also acknowledge that among those children who were classified as not meeting the PA guidelines, some of them may have practised PA for at least 60 min on one day while others may have practised PA on 6 days. Their profiles related to PA are different, and these might be considered when interpreting the results of the present study. Therefore, future studies might also explore diverse behavioural profiles based on the other cut-off points of each of the 24-h Movement behaviours.

Regarding the measures and instruments aligned with Tapia et al.^7^, a more harmonised device-based PA, sedentary behaviour and sleep duration are needed in future research. Furthermore, the present study only measured and discriminated the results of weekdays and weekend days for sleep. Future studies should explore, analyse and compare the adherence to the 24-h Movement Guidelines between weekdays and weekend days since adopting these behaviours can be different^7,34,38^. This is the first study that uses a large sample to characterise the adherence to 24-h Movement Guidelines in Portuguese children aged 6–9. A major strength is the large sample of children aged 6–9 years in a study like this in Portugal. Further cross-sectional, longitudinal, and intervention studies are needed to advance knowledge in promoting the adoption of 24-h Movement Guidelines among children to benefit their health and quality of life.

## Conclusion

This research has shown that only 3.7% of children met all three 24-h Movement Guidelines, whereas 11.9% of children met none of the three guidelines. Likewise, it is highlighted that no significant differences have been found in compliance with the 24-h movement period based on sex. Although compliance and non-compliance with the three 24-h Movement Guidelines were higher in boys than in girls, no significant differences were found. Finally, public policies to promote the three-movement behaviours are recommended to work integrally and allow the positive consequences that this entails to be achieved.

## Data Availability

The datasets generated and analysed during the current study are private because the participants need to give written consent for their data to be shared publicly. Data are however available from the authors upon reasonable request and dependent on the authorisation of the Sintra Grows Healthy Consortium Scientific Leadership.
